# Effects of Cell Differentiation on the Phagocytic Activities of IgM^+^ B Cells in a Teleost Fish

**DOI:** 10.3389/fimmu.2019.02225

**Published:** 2019-09-19

**Authors:** Liting Wu, Linghe Kong, Yanjian Yang, Xia Bian, Siwei Wu, Bingxi Li, Xiaoxue Yin, Liangliang Mu, Jun Li, Jianmin Ye

**Affiliations:** ^1^Guangdong Provincial Key Laboratory for Healthy and Safe Aquaculture, Institute of Modern Aquaculture Science and Engineering, School of Life Sciences, South China Normal University, Guangzhou, China; ^2^School of Biological Sciences, Lake Superior State University, Sault Ste. Marie, MI, United States

**Keywords:** IgM^+^ B cells, *Oreochromis niloticus*, phagocytosis, phagocytic capacity, phagocytic ability

## Abstract

Teleost B cells have phagocytic activities for ingesting particulate antigens, such as bacteria, in addition to the functional secretion of immunoglobulins (Igs). In the present study, the phagocytic activities of IgM^+^ B cells under various differentiational conditions residing in peripheral blood leukocytes were investigated in a teleost fish Nile tilapia (*Oreochromis niloticus*). The IgM^+^ B cells were recognized as IgM^lo^ or IgM^hi^ subsets based on their membrane IgM (mIgM) levels. The mIgM, secreted IgM (sIgM), major histocompatibility complex class II and reactive oxygen species were detected. Expressions of transcription factors (Pax5 and Blimp-1) and B cell signaling molecules (CD79a, CD79b, BLNK, and LYN) suggested that IgM^lo^ B cells were resembling as plasma-like cells and IgM^hi^ resembling as naïve/mature B cells, respectively. Analysis of phagocytic activities demonstrated that both IgM^lo^ and IgM^hi^ B cells have a similar phagocytic ability (phagocytosis percentage); however, the phagocytic capacity [phagocytic index and the mean fluorescence intensity (MFI)] of IgM^hi^ B cells was significantly higher than that of IgM^lo^ B cells. Taken together, the results indicated that B cell differentiation may cause the decrease of phagocytic capacity but not phagocytic ability of phagocytic IgM^+^ B cells in teleost. The finding may provide an evolutionary evidence for understanding the greater specialization of the B cell in more sophisticated adaptive humoral immunity, by decreasing phagocytic activity in order to contribute its function more specifically into antibody-secreting.

## Introduction

Phagocytosis is an important defense against pathogen infection in innate immunity, which is a bridge linking innate immunity and adaptive immunity as well (1). Its characteristics mainly include the large particulates endocytosis (diameter >0.5 μm), actin polymerization and remodeling, phagolysosome formation (2). Macrophages, monocytes, granulocytes, and dendritic cells are generally considered as professional phagocytes. B cells are antibody (Ab)-producing cells, which can produce immunoglobulins (Igs) to specifically neutralize certain antigens, seemed unable to phagocytose antigenic particles in mammals (3). However, teleost fish B cells, as well as B cells of amphibians (*Xenopus laevis*), were firstly demonstrated to be phagocytic and microbicidal, and their phagocytic function has been characterized similarly as that of professional phagocytes (4). Thereafter, the function of B cell phagocytic ability in teleost has also been identified in many fish species, such as rainbow trout (*Oncorhynchus mykiss*), lumpfish (*Cyclopterus lumpus* L.), Atlantic cod (*Gadus morhua* L.), half-smooth tongue sole (*Cynoglossus semilaevis*), channel catfish (*Ictalurus punctatus*), and turbot (*Scophthalmus maximus*) (5–9). The study of phagocytosis in teleost fish B cells might facilitate the exploration of new immune therapy to infection diseases in aquaculture.

In mammals, pre-B cell or B cell lines have been demonstrated to be able to differentiate into macrophages (10, 11). Moreover, the phagocytic activities of peritoneal B1 cells rather than B2-B cells were also identified in mice (12). It has been reported that mouse CD5^+^ B cells (named as B1 cells) can differentiate into cells with classical macrophage morphology (13), and the B1 cells have also shown potential innate immunity with production of over 80% of natural serum antibodies (14, 15). B lymphocytes in teleost fish have been considered as functional equivalent to mammalian B1 cells (16, 17), and the recent identification of a homolog of CD5 in rainbow trout provides further solid evidence that the phenotypes and functions of IgM^+^ B cells are similar to mammalian B1 cells (18). Unlike mammalian B1 cells with low affinity and wide reactivity to antigens, teleost B cells differentiate into Ab-secreting cells in response to antigenic stimulation (19). The B cell subsets, differentiate from naïve B cells (expression of membrane-bound Ab) into plasmablasts and then, terminally differentiate into plasma cells (including short-lived plasma cells and long-lived plasma cells) with a stronger Ab-secreting ability and capacity (19–21).

So far, four subsets of B cells have been identified in teleost fish, named IgM^+^/IgD^+^, IgM^−^/IgD^+^, IgM^+^/IgD^−^, and IgT^+^ B cells (5, 7, 22–24). Among them, IgM^+^ B cells are the predominant B cells, with several folds higher mRNA transcriptions of IgM isotype in the peripheral blood leukocytes (PBLs) of teleost fish like salmon and rainbow trout (25, 26), which are specialized in systemic immunity (9, 27). Both IgM^+^ and IgT^+^ B cells have been found to be phagocytic (4, 22); however, the phagocytic activity and other related roles of B cells under various differentiational conditions (including naïve/mature B cells, activated B cells, plasmablasts, and plasma cells) are poorly understood.

Although B cell developmental pathways in teleost fishes are poorly understood as many essential molecular markers are not yet available; however, some very conservative mammalian transcription factors, like paired box-5 (Pax5) and B lymphocyte-induced protein-1 (Blimp-1), have been studied in rainbow trout (28–30). Pax5 is expressed from the pre-B cell through mature B cells, downregulated during terminal differentiation and absent at the plasma cells in rainbow trout (28). In contrary to Pax5, Blimp-1 is a master regulator of cell differentiation including both terminal B cell and macrophage differentiation (29). Blimp-1 shifts Ig expression from the membrane to the secreted form, leading to increases in secreted Ig (sIg) expression in activated B cells with simultaneous reduction of mIg (29). A recent study demonstrated that the more differentiation of teleost B cells, the less expression membrane IgM (mIgM), and the related B cells, with low or high mIgM expression levels, could be divided into IgM^lo^ or IgM^hi^ subtypes, respectively (30). What's more, the transcription levels of various B cell signaling molecules changed in the process of B cell differentiation, such as CD79a, CD79b, BLNK, and LYN, which were down-regulated during B cell maturation (31, 32).

In this report, we aimed to gain better understanding of B cell phagocytosis in teleost under various differentiational status, in particular for both IgM^hi^ and IgM^lo^ B cell subsets, which are resembling as naïve/mature B cells and plasma-like B cells, respectively. For this purpose, the phagocytic efficiency of Nile tilapia (*Oreochromis niloticus*) IgM^+^ B cells ingesting fluorescent microspheres and killed pathogen *Streptococcus agalactiae* (*S. agalactiae*) were elucidated through flow cytometric analysis, respectively. Our results suggested that only the phagocytic capacity, but not phagocytic ability was significantly affected by the differentiation condition of teleost B cells. This finding shed new lights on the avenue to better understand the functional roles of B cells in the evolutionary process from teleost fish to mammalian species.

## Materials and Methods

### Fish

Healthy Nile tilapias (*Oreochromis niloticus*) with mean weight of 750 ± 50 g were obtained from Guangdong Tilapia Breeding farm (Guangdong, China). All fish were maintained in 300 L tanks in the laboratory with recirculating pre-treated (biologically filtered, dechlorinated, chemically balanced, and UV-treated) fresh water. Water temperature was maintained at 28 ± 2°C, and photoperiod was adjusted to match seasonal change (33, 34). All fish experimental procedures were reviewed and the ethics were approved by the University Animal Care and Use Committee of the South China Normal University.

### Isolation of Peripheral Blood Leukocytes

The total peripheral blood leukocytes were prepared according to the previous procedure with some modifications (20, 28). Nile tilapia were anesthetized in water containing ~0.04% MS-222 (Aladdin, China). Blood samples were collected by venipuncture from the caudal vein with a heparinized syringe [250 μL heparin (0.1 g/L) in 2.5 mL syringe]. Each fish was collected 5 mL blood and then placed in sterile 10 mL tube. After centrifuged at 500 × g for 15 min, the plasma was removed. The blood cells were resuspended gently in four times RPMI-1640 medium (Gibco, USA), supplemented with 100 I.U./mL penicillin G, 100 μg/mL streptomycin, 10 units/ mL heparin and 5% fetal bovine serum (FBS) (Gibco, USA), and then put on ice. The diluted blood cells were then layered upon an equal volume of Histopaque 1077 (Sigma, USA) in 50 mL conical centrifuge tubes slowly, 500 × *g* centrifuged for 40 min at 4°C. Leukocytes were collected from the interface layer, and then washed three times with RPMI-1640 by centrifugation. Cell viability was determined by 0.4% (Sigma, USA) trypan blue staining, and finally peripheral blood leukocytes (PBLs) were resuspended to a concentration of 1 × 10^7^ cells/mL in RPMI-1640 containing 10% FBS.

### Immunofluorescence Staining of PBLs

For immunofluorescence staining, the PBLs were suspended with phosphate buffer saline (PBS, pH = 7.4) and then incubated with mouse anti-IgM monoclonal antibody (mAb, IgG1 type from Balb/c mice) labeled by Alexa Fluor 647 (AF647) (35, 36) for 1 h at room temperature (RT). After washing three times with PBS, the cells were incubated with 1 μg/mL of DAPI (Sigma, USA) for 10 min. Then the cells were washed again with PBS and subjected to microscopy observation (Zeiss, Germany). As a negative control, an isotype mouse IgG was also applied for the above staining procedure.

### Flow Cytometry (FACS)

The isolated PBLs were incubated with AF647-labeled mouse anti-Nile tilapia IgM mAb (1 mg/mL, 1:2000 dilution) at RT for 1 h (35, 36). After washing with PBS, cells were resuspended in RPMI-1640 contained 5% FBS and subjected to FACS analysis with a BD Arial III flow cytometer (BD, USA) and 50,000 cells were recorded in each sample. PBLs incubated without any antibody or with a normal isotype mouse IgG (Thermo, USA) were also used as blank or negative controls. Further data analysis was performed using FlowJo X.

### Cell Sorting

PBLs were incubated with mouse anti-Nile tilapia IgM mAb (35, 36) as described above and only the gated lymphocyte-like cells were selected for sorting in a BD FACS Aria III flow cytometer based on the low forward scatter (FSC) and sideward scatter (SSC) profiles (to exclude the granulocytes). According to the different fluorescence intensity, IgM^−^, IgM^hi^, IgM^lo^, and total IgM^+^ B cells were collected. The purity of various sorted cell populations was analyzed (**Figure 2A**). The sorted cells showing a higher purity level (>95%) were collected in Trizol reagent (Vazyme, China) and immediately frozen by liquid nitrogen, and then stored at −80°C for further isolation of total RNAs.

### Gene Expression Analysis

Total RNA was extracted using Trizol reagent kit (Vazyme, China) according to the manufacture's instruction, and their quality and quantity was determined by Nanodrop 2000 assay (Thermo, USA). The cDNAs were synthesized from the purified RNA and then diluted 10-fold, and stored at −80°C for further quantitative real time PCR analysis (qPCR). For characterization of various B cell subsets, the transcription levels of membrane IgM (mIgM), secreted IgM (sIgM), major histocompatibility complex class IIβ (MHC IIβ) (37), transcription factors (Pax5 and Blimp-1), and B cell signaling molecules (CD79a, CD79b, BLNK, and LYN) were investigated using the 7500 Real Time PCR System (Applied Biosystem, USA) with the SYBR green dye method in a total of 20 μL volume containing 10 μL of 2 × SYBR mix (Yeasen, China), 2 μL forward primer and 2 μL reverse primer, 3 μL of diluted cDNA, 3 μL double distilled H_2_O. The β-actin (Accession No. KJ126772.1) gene was used as internal control with primers showed in Table 1. Gene-specific primers are listed in Table 1. The qPCR was carried out with the following program: 95°C for 3 min, followed by 40 cycles of 95°C for 15 s, 60°C for 1 min.

**Table 1:** Primes used for qPCR in this study.

**Gene**	**Primer**	**Sequence**
mIgM	qmIgM-F	5′-ACTTGATTGAGCCTTTGAGGGAACC-3′
	qmIgM-R	5′-GACTCATGGTTAAAATGCAGAATGC-3′
sIgM	qmIgM-F	5′-ACTTGATTGAGCCTTTGAGGGAACC-3′
	qmIgM-F	5′-GACTCATGGTTAAAATGCAGAATGC-3′
CD79a	qCD79a-F	5′-CATCATAACAAAACTCAGGAGG-3′
	qCD79a-R	5′-GTAGACACGCAGGTAGGTTCCAT-3′
CD79b	qCD79b-F	5′-TGTGCCCATTTACTGTTCATCCTC-3′
	qCD79b-F	5′-CGCCACTGTGCCTCATTTGT-3′
BLNK	qBLNK-F	5′-CCTCCCCAAAGCCTCCTGAA-3′
	qBLNK-R	5′-GCGAAACAAGGCATCGTCAG-3′
LYN	qLYN-F	5′-GATGCCTCAGCCCGACAACT-3′
	qLYN-R	5′-TGTCCCTCTGTGGCGGTGTA-3′
Pax5	qPax5-F	5′-AGGAAGCATCAGACCCG-3′
	qPax5-R	5′-ATCTCCCAGGCGAACA-3′
Blimp-1	qBlimp-1 F	5′-AAGACGCCAACCGCAAAT-3′
	qBlimp-1 R	5′-GAGTGGGCTGGGTTCACATAC-3′
MHC IIβ	qMHC IIβ F	5′-ACTGACTGGGACCCGTCCAT-3′
	qMHC IIβ R	5′-ACAGGAAGCAGCCGCTTTTA3′
β-actin	qβ-actin F	5′-CAAAGCCAACAGGGAGAA-3′
	qβ-actin R	5′-CTTGATGTCACGCACGAT-3′

*F, forward; R: reverse*.

### The Comparation of sIgM Secreted From IgM^hi^ and IgM^lo^ Cells at Protein Level

In order to compare the antibody secreting abilities of IgM^hi^ and IgM^lo^ B cells at protein level, a previous published ELISA was explored (36). Briefly, IgM^hi^ and IgM^lo^ B cells (1 × 10^6^ cells per sample) were sorted (as the description of “Cell Sorting”) and cultured in RPMI-1640 medium (Gibco, USA), supplemented with 100 I.U./mL penicillin G, 100 μg/mL streptomycin, and 10% FBS (Gibco, USA) for 24 h. The culture supernatants were collected and sorted in −20°C. Microtiter plate (Corning, USA) was coated with 2 μg/mL mouse anti-tilapia IgM mAb (100 μL) at RT for 1 h. Then, the plate was washed three times with 1 × TTBS (contained 0.1% Tween 20), and blocked with 200 μL 0.5% BSA-TTBS for 1 h at RT. Plate was then washed three times with 1 × TTBS. Cell supernatant was added to well, 100 μL per well and incubated for 1 h at RT. Followed by three times washes (1 × TTBS), 100 μL of biotinylated mouse anti-tilapia Ig mAb (0.25 μg/mL) (36) in blocking buffer were then added, and incubated for 1 h at RT. After three times washes, 100 μL of streptavidin-HRP (0.5 μg/mL) (Southern Biotech, USA) was added, and incubated for 1 h at RT. After washed three times, 2,2′-Azinobis-(3-ethylbenzthiazoline-6-sulphonate) (ABTS) (Sigma, USA) of 100 μL was added for 10 minuses, and the optical density (O.D.) was measured using a microplate reader (Thermo, USA) at 405 nm. The ratio of the O.D. value of IgM^lo^ sample to IgM^hi^ sample were taken as the relative expression of sIgM at protein level.

### Reactive Oxygen Species (ROS) Measurement

ROS levels of IgM^hi^ and IgM^lo^ cells were determined using ROS assay kit (Beyotime, China). Cells (1 × 10^6^) were obtained as the description of “Isolation of Peripheral Blood Leukocytes,” were treated with 10 μM DCFH-DA dissolved in PBS (1 mL) at 25°C for 1 h. The control sample was the same amount cells without fluorescent dyes. The fluorescence intensity was monitored with excitation wavelength at 488 nm and emission wavelength at 530 nm (38). The mean fluorescence intensity (MFI) was analyzed using FlowJo X.

### Transmission Electron Microscopy (TEM)

FACS sorted cell fractions (IgM^−^, IgM^lo^, and IgM^hi^ cells) were fixed in 2.5% glutaraldehyde in PBS (pH 7.4) and prepared for TEM analysis according to a previous method (7). Cell images were observed and recorded by using the transmission electron microscope Tecnai (FEI, USA).

### Assessment of Phagocytosis

A total of 12 individual fish (750 ± 50 g) were used to investigate the phagocytic activity of IgM^+^ lymphocytes in Nile tilapia. PBLs were adjusted to 1 × 10^7^ cells/mL using RPMI-1640 medium supplemented with 5% FBS. PBLs were incubated with 0.5 and 1 μm Fluoresbrite® YG carboxylate microspheres (YG beads) (Polysciences Inc., USA) at a 1:20 (cells: beads) ratio for 4 h at 25°C, respectively (4, 9). For phagocytosis of bacteria, the predominant pathogens *S. agalactiae* was used here. The inoculation, bacterial counting, inactivation and fluorescein isothiocyanate (FITC; Sigma, USA) labeled modes of *S. agalactiae* were performed as described by our previous reports (34, 39). The ratio of cells vs. bacteria for phagocytosis was 1:20 for 4 h at 25°C as well. After incubation, the cells were collected and centrifuged at 100 × g for 10 min at 4°C to remove excess beads. Then the cells were resuspended in 1 mL PBS containing 5% FBS, and incubated with anti-IgM mAb labeled with AF 647 (1 mg/mL, 1:2000 dilution) as described above (35). After three times washes with PBS, the phagocytic activities of PBLs from 14 fish were independently analyzed by using BD Arial III flow cytometer (BD, USA). PBLs incubated without any antibody or with a normal isotype mouse IgG (Thermo, USA) were also included as blank or negative controls. Phagocytic activities of IgM^+^ cells were expressed as phagocytic ability (% of total phagocytic cells that ingested one or more beads) and phagocytic capacity (the proportion of phagocytic cells that had ingested one, two or three or more beads, respectively), as well as the MFI (6, 7, 40). Data analyses were performed using FlowJo X.

### Statistical Analysis

Statistical analysis was carried out by using SPSS 17.0 software (SPSS, USA). Data were analyzed with analysis of variance (ANOVA) followed by two-tailed Student's *t*-test when the ANOVA indicated that the variances of both groups differed significantly. No significant difference (n.s.) means *p* > 0.05 and significant difference was defined as *p < 0.05, **p < 0.01, and ***p < 0.001.

## Results

### IgM^+^ B Cells in PBLs

**Figure 1:**
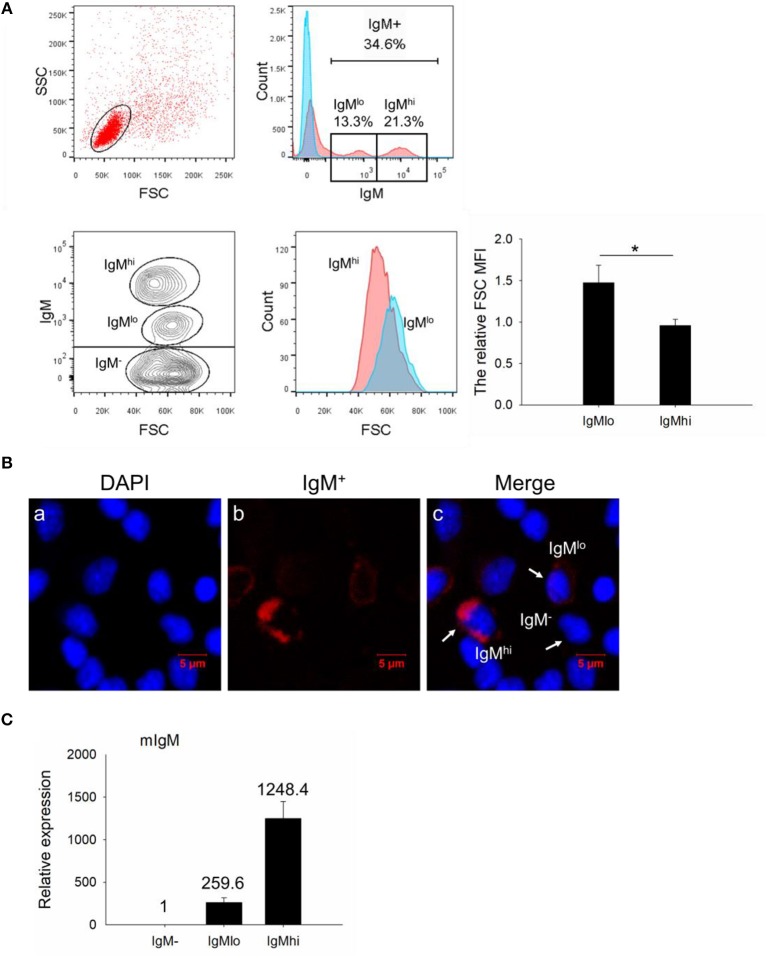
FACS analysis, immunofluorescence staining, and expression profiles of mIgM in isolated leukocytes. **(A)** In the upper panel, a representative scatter plot of PBLs were gated with lower FSC/SSC cell populations (lymphocytes gate) for further IgM^+^ B cells analysis. The corresponding analysis of IgM^+^ cells labeled with AF647 were defined as red shadow line, while the control samples were in blue shadow lines in the histogram. In the lower panel, the size of IgM^hi^ and IgM^lo^ cells were analyzed by the FSC in the scatter plot and histogram. The difference between IgM^hi^ and IgM^lo^ was shown as the relative FSC MFI in IgM^lo^ compared to IgM^hi^ cells. **(B)** IgM^−^, IgM^hi^, and IgM^lo^ B cells were visualized under fluorescence microscope: IgM^+^ cells labeled with red fluorescence; nuclei stained with DAPI showed blue fluorescence. **(C)** Expressions of mIgM on IgM^−^, IgM^lo^, and IgM^hi^ cells. Data are shown as the mean relative expression to the transcription of the mIgM in IgM^−^ cells. The relative FSC MFI was quantified and shown as bar plots as mean ± SD (*n* = 12, three independent experiments and four individual fish per trial). Statistical differences were evaluated by a one-way ANOVA followed by two-tailed Student's *t*-test, where *means *p* < 0.05.

In order to investigate the IgM^+^ B cells in the PBLs of Nile tilapia, we firstly gated the lymphocyte-like cell population based on their lower FSC and SSC patterns (Figure 1A, upper left panel). Among the gated cell population, a total of 34.6% cells were detected as IgM^+^ B cells, and the histogram also showed two distinct populations, which were designed as IgM^hi^ (21.3%) and IgM^lo^ B cells (13.3%), respectively (Figure 1A, upper right panels). To further identify these two subsets, we analyzed the cell size (according to the FSC parameter) difference between IgM^lo^ and IgM^hi^ cells, which exhibited a significant larger size in IgM^lo^ than IgM^hi^ cells (Figure 1A, lower panels). Under the fluorescence microscope, an obvious variety of red fluorescence intensity colocalized around the surface of IgM^+^ B cells were observed which represented the negative, low or high expressions of IgM on the surface of lymphocytes (Figure 1B). What's more, the transcription level of mIgM was detected in IgM^−^, IgM^lo^, and IgM^hi^ cells as well, which expressed highest in IgM^hi^ cells but lowest in IgM^−^ cells (Figure 1C).

### Cell Sorting and the Ultrastructure of the Sorted Cells

**Figure 2:**
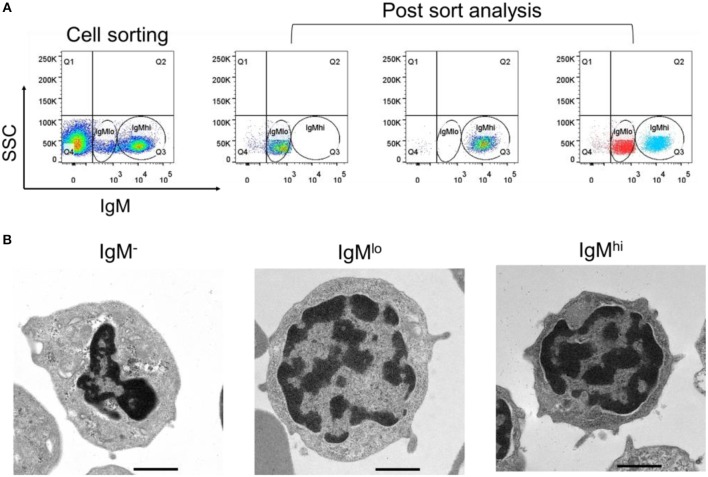
Cell sorting and ultrastructural analysis of IgM^−^, IgM^lo^, and IgM^hi^ cells. **(A)** Left panel of the IgM^hi^ and IgM^lo^ gate was set as a representative PBL sample for sorting. Right panels were the purity check for post sorted IgM^hi^ and IgM^lo^ cells. **(B)** Ultrastructure of IgM^−^ (left panel), IgM^lo^ (middle panel), and IgM^hi^ cells (right panel). Representative cells were magnified 5,000-folds. Scale bar = 1 μm.

For further characterization of these two different IgM^+^ B subpopulations, IgM^hi^ and IgM^lo^ B cells, as well as IgM^−^ cells were sorted out from PBLs according to the difference of fluorescence intensity (Figure 2A, left panel). The purity of sorted IgM^hi^ and IgM^lo^ cells was double checked and a high sorting effectiveness was validated (Figure 2A, right panel). In addition, the ultrastructure of the sorted cells was observed under the transmission electron microscope, both IgM^lo^ and IgM^hi^ cells showed typical lymphocyte features (e.g., large nucleus, thin cytoplasm), and contained a few cytoplasmic vacuoles, however, more abundant cytoplasmic vacuoles were observed in the IgM^−^ cells (Figure 2B).

### Profiles of mIgM, sIgM, MHC IIβ, and ROS on IgM^+^ B Cell Subpopulations

**Figure 3:**
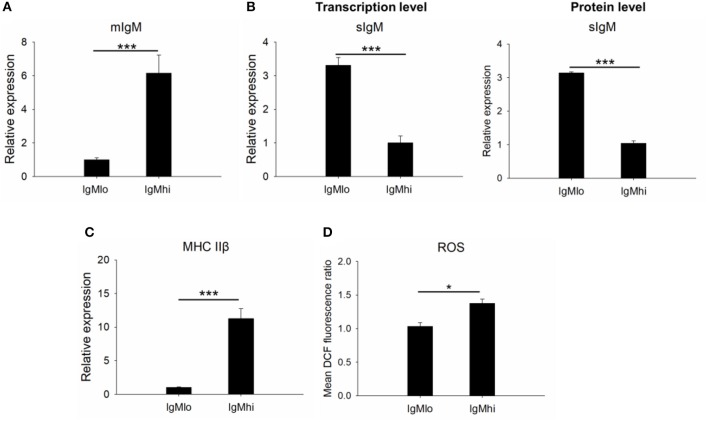
Identification of IgM^hi^ and IgM^lo^ B cell subpopulations. For mIgM **(A)** and MHC II **(C)**, the mean relative expression on IgM^hi^ to the transcription in IgM^lo^ cells was set. The mean relative expressions of sIgM on IgM^lo^ cells compared to IgM^hi^ cells (at transcription level and protein level) were detected **(B)**. The ROS **(D)** on IgM^hi^ and IgM^lo^ B cell subpopulations were detected and showed as the mean DCF fluorescence. Results were shown as mean ± SD (*n* = 12, three independent experiments and four individual fish per trial). Statistical differences were evaluated by one-way ANOVA followed by two-tailed Student's *t*-test. Statistically significant difference was defined as: *means *p* < 0.05; ***means *p* < 0.001.

In order to identify the difference between these two subpopulations, the expressions of mIgM, sIgM, and MHC IIβ by qPCR with specific primers were analyzed (Table 1). In comparison to the IgM^lo^ cells, the expression of *mIgM* in IgM^hi^ B cells was significantly higher than that in the IgM^lo^ subpopulation, and the ratio of IgM^hi^/IgM^lo^ was 6.2 (Figure 3A). In contrast, the level of *sIgM* expressed more than three times higher in IgM^lo^ B cell subpopulation than that in IgM^hi^ B cells (Figure 3B), both at transcription level and protein level. The MHC IIβ displayed significantly higher on IgM^hi^ B cells than that on IgM^lo^ B cells (Figure 3C). DCFH-DA-dependent analysis showed that intracellular ROS levels were significant higher in IgM^hi^ than IgM^lo^ B cells (Figure 3D).

### Expressions of Transcription Factors and B Cell Signaling Molecules

**Figure 4:**
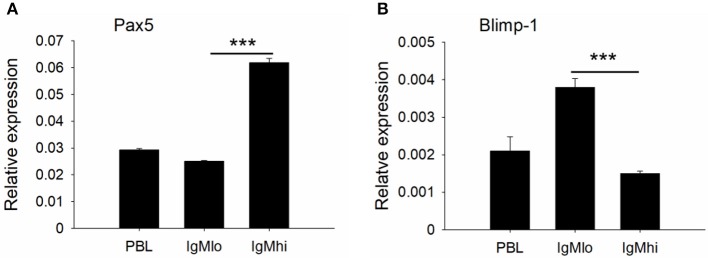
Expression profiles of transcription factors Pax5 **(A)** and Blimp-1 **(B)** on IgM^hi^ and IgM^lo^ B cell subpopulations. The expressions were relative to the endogenous control gene β-actin. Results were shown as mean ± SD (*n* = 12, three independent experiments and four individual fish per trial). Statistical differences were evaluated by one-way ANOVA followed by two-tailed Student's *t*-test. Statistically significant difference was defined as: ***means *p* < 0.001.

**Figure 5:**
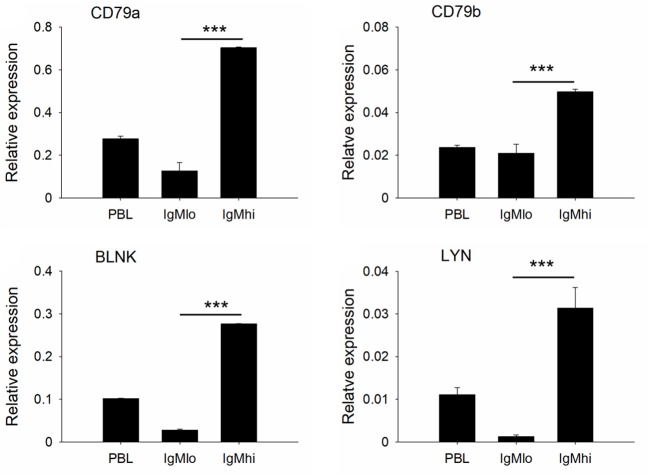
Expressions of B cell signaling molecules decreased in the process of B cell differentiation. B cell signaling molecules (CD79a, CD79b, BLNK, and LYN) relative to the endogenous control gene β-actin were calculated for each subpopulation. Results were shown as mean ± SD (*n* = 12, three independent experiments and four individual fish per trial). Statistical differences were evaluated by one-way ANOVA followed by two-tailed Student's *t*-test. ***means *p* < 0.001.

To further distinguish IgM^hi^ and IgM^lo^ B cell populations, we analyzed the expression profiles of two key B cell transcription factors, Pax5 and Blimp-1, which are related to plasma cell differentiation, as well as a couple of B cell signaling molecules, like CD79a, CD79b, BLNK, and LYN with specific primers (Table 1). The expression profiles for these molecules exhibited significant difference between IgM^hi^ B cells and IgM^lo^ B cells (Figures 4, 5). Transcription of Pax5 was detected significantly high in IgM^hi^ B cells, but low in IgM^lo^ or PBLs (Figure 4A); whereas, the expression level of Blimp-1 was significantly high in IgM^lo^ subpopulation than that in IgM^hi^ subpopulation (Figure 4B). The expression profiles of B cell signaling molecules (CD79a, CD79b, BLNK, and LYN) showed similar patterns in these two subsets, where they all expressed higher levels in IgM^hi^ B cells and lower in IgM^lo^ subset (Figure 5). Therefore, the IgM^hi^ cells and IgM^lo^ cells may represent naïve/mature B cells and plasma-like B cells, respectively.

### Phagocytic Activity of IgM^hi^ and IgM^lo^ B Cells

**Figure 6:**
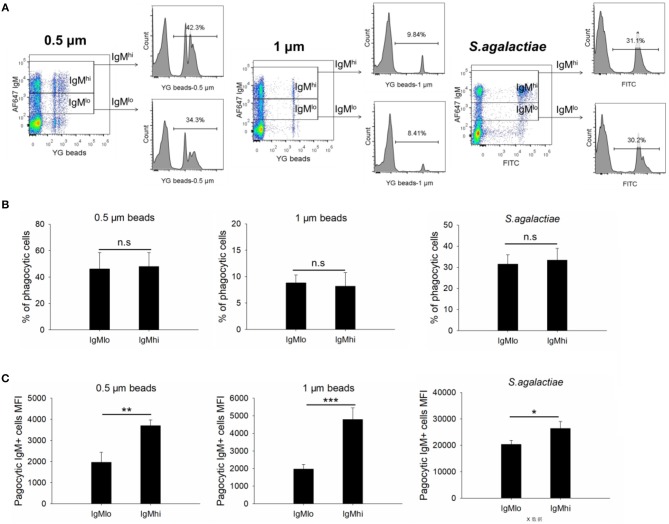
Phagocytic activities of IgM^hi^ and IgM^lo^ B cells. **(A)** Scatter plot and histogram of phagocytic IgM^+^ cells ingested 0.5/1 μm or killed *S. agalactiae*. **(B)** The total average % of phagocytic cells in IgM^hi^ and IgM^lo^ subpopulation. **(C)** The MFI of phagocytic IgM^hi^ and IgM^lo^ B cells. Results were shown as mean ± SD (*n* = 14, three independent experiments and four-five individual fish per trial). Statistical differences were evaluated by a one-way ANOVA followed by two-tailed Student's *t*-test, where n.s means *p* > 0.05, *means *p* < 0.05, **means *p* < 0.01 and ***means *p* < 0.001.

**Figure 7:**
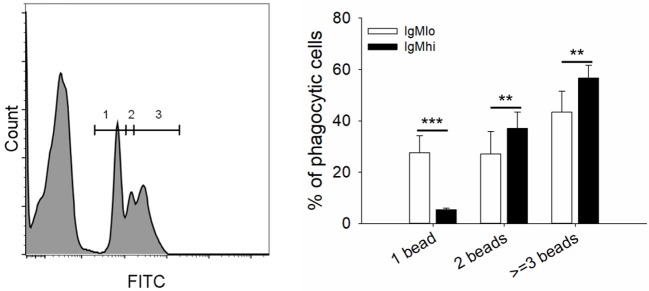
Phagocytic capacity of IgM^hi^ and IgM^lo^ B cells ingested 0.5 μm YG beads. Histogram of phagocytic IgM^+^ cells were divided into three peaks, including 1, 2, and 3, which represented one, two, three or more beads ingested by IgM^+^ cells. And the comparison of phagocytic rate (%) of IgM^hi^ and IgM^lo^ B cells with ingestion of 1, 2, and 3 or more beads. Results were shown as mean ± SD (*n* = 14, three independent experiments and four-five individual fish per trial). Statistical differences were evaluated by a one-way ANOVA followed by two-tailed Student's *t*-test, where **means *p* < 0.01 and ***means *p* < 0.001.

To evaluate the phagocytic activity of IgM^hi^ and IgM^lo^ B cells, the PBLs were incubated with 0.5/1 μm YG fluorescent beads and killed *S. agalactiae*, respectively. Then we analyzed the IgM^+^ cells by FACS. The scatter plot and the histogram of IgM^+^ cell (IgM^hi^ and IgM^lo^) phagocytized fluorescent microspheres or killed pathogen were shown in Figure 6A. When calculated the phagocytic percentage (phagocytic ability) in IgM^lo^ and IgM^hi^ B cells, it performed that the mean percentage of phagocytosis of both IgM^lo^ and IgM^hi^ B cells exhibited similar phagocytic ability to ingest 0.5/1 μm YG fluorescent beads and killed *S. agalactiae* (Figure 6B). For further examination of the phagocytic capacity of IgM^hi^ and IgM^lo^, the MFI of FITC in phagocytic IgM^hi^ and IgM^lo^ subsets were evaluated. As shown in Figure 6C, in comparison to IgM^lo^ B cells, IgM^hi^ B cells showed significantly higher MFI. It was clear that there existed different peak of FITC intensity in the histogram of phagocytic IgM^+^ B cells when ingested 0.5 μm as shown in the Figure 6A. The peaks were divided into 1, 2, and 3 of FITC intensity in the histogram of phagocytic IgM^+^ B cells, which represented the IgM^+^ cells ingesting one bead, two beads, and three or more beads, respectively (Figure 7, left panel). The results indicated that the percentage of IgM^hi^ B cells ingesting two or three and more beads were significantly higher than that of IgM^lo^ B cells, but the percentage of IgM^lo^ cells ingested one bead was higher than IgM^hi^ cells (Figure 7, right panel).

## Discussion

The analysis of teleost B cell subpopulation has been limited because of the lack of antibodies. Using established mAbs against mIgM to distinguish different B cell subsets based on the different fluorescence intensity (41). In this study, we presented the identification of two IgM^+^ B cell subpopulations, IgM^lo^, and IgM^hi^, from PBLs in Nile tilapia based on mIgM expression on cell surface. The IgM^hi^ and IgM^lo^ cells were resembled to be naïve/mature B cell and plasma-like B cell, respectively, according to their expression levels of mIgM, sIgM, MHC IIβ, Pax5, Blimp-1, CD79a, CD79b, BLNK, and LYN molecules. The IgM^lo^ and IgM^hi^ B cells had no significant difference in their phagocytic ability, but the phagocytic capacity in IgM^hi^ cells was significantly higher than that in IgM^lo^ B cells. These results collectively indicated that B cell differentiation may cause the decrease of phagocytic capacity but have no effect on their phagocytic ability in teleost fish.

In our present study, IgM^+^ B cells were demonstrated as the most dominant leukocytes (about 35%) in the PBLs of Nile tilapia (Figure 1A, upper panel), which is comparable to other fish species such as rainbow trout (4, 27). What's more, the FACS analysis clearly revealed that there are two distinct IgM^+^ B subpopulations, IgM^lo^ and IgM^hi^ B cells, which were also clearly visualized under the microscopy for the cells with different fluorescence intensity colocalized on their surface (Figure 1B). In mammals, the expressions of mIgM in differentiated B cell subsets were different, representing higher in naïve B cells but low in plasma cells, and such differentiation and maturation of mammalian B cells was coordinated by the transcription factors of Pax5 and Blimp-1 (42–44). Similar findings of B cell differentiation and their relation to the IgM maker and transcription factors have also been demonstrated in rainbow trout (28). We firstly isolated both IgM^lo^ and IgM^hi^ B cells through cell sorting (Figure 2A) and analyzed by TEM (Figure 2B). The ultrastructural images characterized that IgM^lo^ and IgM^hi^ cells had higher nucleus to cytoplasm ratio than in IgM^−^ cells, which were similar to the typical features of IgM^+^ and IgM^−^ cells in rainbow trout (4, 22). The IgM^+^ (IgM^hi^ and IgM^lo^) B cells were characterized by a large round nucleus, a thin cytoplasm, and a varying number of small dendrites extending from the cells. Significant difference existed between IgM^hi^ and IgM^lo^ B cells in size, which characterized as that the IgM^lo^ was larger than the IgM^hi^ B cell. It was consistent with the scatter plots results of the IgM^hi^ and IgM^lo^ cells in Figure 1B. Moreover, significant higher transcriptions of mIgM were only identified in IgM^hi^ B cells, while sIgM only from IgM^lo^ B cells which indicated the differentiation process of naïve/mature B cells (higher expression of mIgM) (Figure 3A) to IgM-secreting plasma cells (higher expression of sIgM) (Figure 3B). The high level of surface MHC IIβ on IgM^hi^ B cells decreased to IgM^lo^ B cells (Figure 3C), which indicated that the antigen presenting capacity decreased during the process of B cell differentiation. Plasma cells lose their ability to present antigen (45), and results in increased IgM secretion levels (41). A proprietary enzyme system of phagocytes in professional phagocytes is responsible for the production of ROS during respiratory bursts to kill invasive pathogenic microorganisms, and lower ROS level is determined in a mature phagocyte (46). ROS were generated during aerobic metabolism and played important roles as chemical mediators in normal cell growth, differentiation, programmed cell death and senescence (47). Higher level of ROS in IgM^hi^ B cells than that in IgM^lo^ B cells (Figure 3D) indicated that the function in IgM^hi^ B cells may be different with IgM^lo^ B cells. Furthermore, IgM^hi^ B cells were demonstrated to express lower level of Blimp-1 and higher level of Pax5, while IgM^lo^ B cells exhibited higher expression of Blimp-1 and lower expression of Pax5 (Figure 4). These results were in consistent with the findings of the differentiating status of B cells in mammals (48) and in rainbow trout (30), where IgM^hi^ resembled as naïve B cell phenotype and IgM^lo^ as Ab-secreting cell phenotype. The phenomenon of the larger size in IgM^lo^ B cells than in IgM^hi^ B cells (Figure 1A, lower panel; Figure 2B) was consistent with the studies in rainbow trout (41) and in mammals (49). It may imply that in teleost fish, plasma-like cells (IgM^lo^ B cells) build up a large amount of the protein-synthesizing machinery than naïve/mature B cells (IgM^hi^ B cells) as in mammal plasma cells (49). In addition, down-regulation of B cell signaling molecules found in different tilapia IgM^+^ B cell subsets (Figure 5) was also in agreement with mammalian B cells (31, 50), which provides further evidence for the IgM^hi^ and IgM^lo^ B cells represented different differentiating stages of Nile tilapia B cells.

Phagocytic B cells have been identified in various teleost species, and their contribution to fish innate immunity against various pathogens has also been widely accepted (4–9). In responding to *in vitro* stimulations through TLRs (from TLR1 to TLR8), mammalian B1 cells would proliferate and differentiate into Ig-secreting cells (51, 52). Phagocytic fish B cells have been considered as equivalents to mammalian B1 cells because they shared more similarities including the phagocytic function (53); however, until now, it is poorly understood whether cell differentiation has any effect on phagocytic activity in the differentiated B cells in mammals (B1 cells), or in teleost. In the current study, we found that the phagocytic ability (uptake rate in IgM^+^ B cells) had no significant difference between IgM^hi^ and IgM^lo^ B cells (Figure 6B), but the phagocytic capacity in IgM^hi^ was higher than that in IgM^lo^ B cells (Figures 6C, 7). Since IgM^lo^ and IgM^hi^ B cells were B cell subpopulations at different differentiating stages, the findings of the lower of phagocytic capacity in IgM^lo^ than that in IgM^hi^ B cells might indicate the decrease of B cell phagocytic capacity in the process of B cell differentiation in teleost. With the exploration of the phagocytic activity changing during B cell differentiation in teleost, we can better understand the roles of B cell against infection and in bridging innate and adaptive immunity.

**Figure 8:**
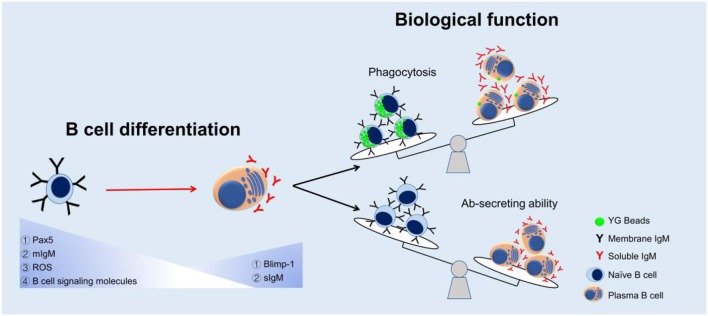
A proposed model of change in B cell function during the B cell differentiation in teleost. Expressions of Pax5, mIgM, ROS, and B cell signaling molecules decreased while Blimp-1 and sIgM expressions increased in the process of B cell differentiation. The phagocytic capacity decreased but Ab-secreting ability increased as a result of B cell maturation.

The appearance of the phagocytic IgM^+^ B cells in teleost supports the idea of an evolutionarily relationship between B cells and macrophages, in which B cells might have evolved from ancient phagocytic cells (1). However, the main activity of fish B cells is considered to produce high content of natural serum IgM molecules in unimmunized fish (54–56), especially when the B cells differentiate into plasma cells (19, 20). According to the current findings, we propose a model of change in B cell function during the B cell differentiation in teleost (Figure 8). We hypothesize that when the B cells differentiate into Ab-secreting cells (plasma cells), the phagocytic capacity decreases so as to contribute these B cells more specialized to provide efficient Abs in more sophisticated adaptive humoral immunity. In mammals, besides B1 B cells, there are other B cell subsets, including marginal zone B cells and follicular B2 cells. B2 B cells can produce Abs with high affinity and specificity to T-dependent antigens but are unable to be phagocytic (57). These B2 B cells would be able to differentiate into plasma cells under the stimulus of pokeweed mitogen. All these findings may indicate that the B cells, in the process of evolution, decrease other functions, such as phagocytosis, in order to specialize its function for Ab-secreting. The decrease of phagocytic capacity in teleost B cell during B cell differentiation, from naïve B cells to terminal differentiating plasma cells (possessing strong Ab-secreting ability), might support evolutionary relationship of mammalian B1 B cells and B2 B cells, and provide more evidence for understanding the greater specialization of these B cells in more sophisticated adaptive humoral immunity in mammals.

## Author Contributions

LW performed most of the experimental work. LK, YY, and SW assisted LW in the preparation of sample and cell sorting. XB and XY provided primers and performed all transcriptional analysis. BL and LM assisted data analysis and graphing. JL reviewed and polished the manuscript. JY and LW designed the experiments and wrote the main body of the paper.

### Conflict of Interest Statement

The authors declare that the research was conducted in the absence of any commercial or financial relationships that could be construed as a potential conflict of interest.
